# Targeting the SMURF2-HIF1α axis: a new frontier in cancer therapy

**DOI:** 10.3389/fonc.2024.1484515

**Published:** 2024-12-04

**Authors:** Emile Youssef, Shuai Zhao, Connor Purcell, Gary L. Olson, Wafik S. El-Deiry

**Affiliations:** ^1^ Research & Development, SMURF-Therapeutics, Inc., Providence, RI, United States; ^2^ Medical & Pharmacovigilance, Kapadi, Inc., Raleigh, NC, United States; ^3^ Department of Pathology & Laboratory Medicine, Legorreta Cancer Center at Brown University, Providence, RI, United States; ^4^ Medicinal Chemistry & Drug Discovery, Provid Pharmaceuticals, Inc., Monmouth Junction, NJ, United States

**Keywords:** SMURF2, HIF1α, tumor microenvironment, ferroptosis, hypoxia, metabolic reprogramming, angiogenesis, cancer therapy

## Abstract

The SMAD-specific E3 ubiquitin protein ligase 2 (SMURF2) has emerged as a critical regulator in cancer biology, modulating the stability of Hypoxia-Inducible Factor 1-alpha (HIF1α) and influencing a network of hypoxia-driven pathways within the tumor microenvironment (TME). SMURF2 targets HIF1α for ubiquitination and subsequent proteasomal degradation, disrupting hypoxic responses that promote cancer cell survival, metabolic reprogramming, angiogenesis, and resistance to therapy. Beyond its role in HIF1α regulation, SMURF2 exerts extensive control over cellular processes central to tumor progression, including chromatin remodeling, DNA damage repair, ferroptosis, and cellular stress responses. Notably, SMURF2’s ability to promote ferroptotic cell death through GSTP1 degradation offers an alternative pathway to overcome apoptosis resistance, expanding therapeutic options for refractory cancers. This review delves into the multifaceted interactions between SMURF2 and HIF1α, emphasizing how their interplay impacts metabolic adaptations like the Warburg effect, immune evasion, and therapeutic resistance. We discuss SMURF2’s dual functionality as both a tumor suppressor and, in certain contexts, an oncogenic factor, underscoring its potential as a highly versatile therapeutic target. Furthermore, modulating the SMURF2-HIF1α axis presents an innovative approach to destabilize hypoxia-dependent pathways, sensitizing tumors to chemotherapy, radiotherapy, and immune-based treatments. However, the complexity of SMURF2’s interactions necessitate a thorough assessment of potential off-target effects and challenges in specificity, which must be addressed to optimize its clinical application. This review concludes by proposing future directions for research into the SMURF2-HIF1α pathway, aiming to refine targeted strategies that exploit this axis and address the adaptive mechanisms of aggressive tumors, ultimately advancing the landscape of precision oncology.

## 1 Introduction

The intricate interactions between cellular survival pathways and the tumor microenvironment (TME) are fundamental drivers of cancer progression and therapeutic resistance (1). While Hypoxia-Inducible Factor 1-alpha (HIF1α) plays a central role in facilitating cellular adaptation to hypoxic conditions within solid tumors, recent insights have revealed that the regulation of HIF1α by SMAD-specific E3 ubiquitin protein ligase 2 (SMURF2) is equally crucial in determining cancer cell fate. HIF1α’s activation and translocation to the nucleus, where it dimerizes with HIF1β and induces the expression of survival and proliferation genes, is well-documented (2). These processes are essential for promoting tumor survival, expansion, and metastasis by enabling cancer cells to thrive under hypoxic conditions. However, the degradation of HIF1α by SMURF2 introduces a regulatory layer that could be exploited therapeutically (3). SMURF2 targets HIF1α for ubiquitination, leading to its proteasomal degradation, which disrupts key adaptive processes such as the Warburg effect—where cancer cells shift towards aerobic glycolysis for rapid ATP production, contributing to an acidic microenvironment that furthers tumor progression and therapy resistance (4). Additionally, while HIF1α regulates the incorporation of glutamine into the tricarboxylic acid (TCA) cycle and lipid synthesis to support anabolic growth (5–7), SMURF2’s role in modulating these pathways through HIF1α degradation highlights its potential as a therapeutic target. By destabilizing HIF1α, SMURF2 not only limits these metabolic adaptations but also impacts the epithelial-mesenchymal transition (EMT), thereby reducing the invasive potential of tumors (8, 9).

SMURF2 has emerged as a significant player that binds to HIF1α, a key regulator of cellular responses to hypoxia, initiating its degradation through a ubiquitination mechanism and thereby regulating its activity. This process influences gene expression associated with angiogenesis, metabolism, and tumor progression, potentially increasing tumor susceptibility to immune-mediated destruction and enhancing responsiveness to conventional therapies. SMURF2’s tumor suppressor properties extend beyond HIF1α regulation. It modulates various cellular processes by targeting and promoting the degradation of key regulatory proteins such as the transcription factors KLF5, ID1/ID3, and YY1 (10, 11). This impacts gene expression, chromatin structure, and the DNA damage response, thereby influencing tumor suppression through p53 activation, c-Myc suppression, and stabilization of the Mad2 protein, which collectively affect cell division and tumorigenesis. In colorectal cancer, SMURF2 ubiquitinates and degrades the carbohydrate response element-binding protein (ChREBP), curbing aerobic glycolysis and cell proliferation. Furthermore, SMURF2 interacts with and degrades sirtuin 1 (SIRT1), with its depletion leading to increased SIRT1 levels, promoting colorectal cancer formation and growth. Elevated SMURF2 mRNA levels have been associated with improved outcomes in clear cell renal cell carcinoma (ccRCC), suggesting its potential as a biomarker for prognosis and a therapeutic target (12, 13).

Moreover, SMURF2’s role in promoting ferroptosis opens novel therapeutic strategies for tumors resistant to standard treatments (1, 14, 15). Alterations in the SMURF2-HIF1α interaction can affect these processes, offering targets to enhance cancer treatment and potentially bypass resistance to existing therapies. Although the SMURF2-HIF1α pathway holds promise for managing cancer cell survival and resistance in the hypoxic TME, its clinical application remains to be developed. No interventions specifically targeting SMURF2 have been developed, and there have been no FDA approvals or advancements in HIF1α beyond early clinical trials. This underscores a significant gap between laboratory findings and practical implementation in real-world settings. This gap is attributed to the pathway’s complex role in molecular resistance, potential side effects, and the regulation of cellular survival mechanisms.

The elucidation of SMURF2’s role in HIF1α regulation opens potential strategies for cancer therapy, particularly in hypoxia-driven tumors where HIF1α plays a key role in tumor aggressiveness, metastasis, and resistance to therapy. By destabilizing HIF1α, SMURF2 disrupts the hypoxic adaptation mechanisms of cancer cells, thereby reducing their survival advantage and possibly enhancing the efficacy of existing treatments (13). Recent insights have highlighted the critical role of SMURF2 in the process of ubiquitination and degradation of HIF1α under both normoxic and hypoxic conditions, a process critical for modulating tumor progression, angiogenesis, and cellular metabolism (6). Specifically, SMURF2’s activity facilitates the maintenance of low intracellular HIF1α levels, impacting cellular responses to hypoxia and inhibiting tumor growth and metastasis. Additionally, SMURF2’s influence extends beyond HIF1α, affecting chromatin condensation, DNA damage response, and the stability of other proteins involved in tumorigenesis, such as RNF20. These findings highlight SMURF2’s broad regulatory impact and its potential as a therapeutic target in cancer treatment (12, 13).

While HIF1α is a well-established factor in cancer biology, this review article focuses on the intricate interplay between SMURF2 and HIF1α. Specifically, SMURF2’s role as an E3 ubiquitin ligase in regulating HIF1α stability and activity is central to its function in cancer. Recent studies have demonstrated that SMURF2 mediates the ubiquitination and subsequent degradation of HIF1α, thereby modulating the cellular response to hypoxia—a key driver of tumor progression and metastasis. This interaction suggests that SMURF2 is not only a regulator of HIF1α but also a potential therapeutic target in cancers where hypoxia plays a critical role. Furthermore, this review aims to elucidate the intricate interplay between HIF1α, SMURF2, and the TME, shedding light on their significance in shaping tumorigenesis and cancer progression. Subsequent sections will comprehensively discuss this topic.

## 2 Overview of SMURF2 and HIF1α roles in cancer biology

SMURF2 and HIF1α are pivotal regulators in cancer biology, influencing pathways associated with protein degradation, hypoxic response, and cellular adaptation. SMURF2, an E3 ubiquitin ligase, mediates the ubiquitination and degradation of several cancer-related proteins, modulating signaling pathways that affect tumor growth, migration, and metastasis. HIF1α, as a transcription factor, enables cellular adaptation to low oxygen levels, a common trait in solid tumors, by activating genes related to angiogenesis, metabolic reprogramming, and survival. Examining the roles of SMURF2 and HIF1α in different cancers reveals their complex contributions to tumor progression and potential as therapeutic targets. SMURF2’s role in cancer is context-dependent, acting both as a tumor suppressor and, paradoxically, as an oncogene in certain cancers. As a tumor suppressor, SMURF2 can inhibit cell proliferation and prevent malignant transformation. However, in specific cancer subtypes, SMURF2’s activity promotes oncogenic pathways, such as the Wnt/β-catenin signaling pathway. By targeting negative regulators for degradation, SMURF2 enhances β-catenin activity, which can drive tumor progression, particularly in cancers where Wnt signaling is aberrantly active (16, 17).

In ovarian cancer, SMURF2 acts on RACK1, an adaptor protein involved in cancer signaling. SMURF2 ubiquitinates RACK1, marking it for degradation. Loss of SMURF2 stabilizes RACK1, promoting cancer cell survival and proliferation. Elevated RACK1 levels in the absence of SMURF2 correlate with poor patient outcomes, positioning SMURF2 as a critical regulator in ovarian cancer progression (18). In breast cancer, SMURF2 functions predominantly as a tumor suppressor, and its expression is frequently downregulated in triple-negative breast cancer (TNBC), contributing to the aggressiveness of this subtype. Mechanistically, SMURF2’s downregulation appears to be mediated by specific microRNAs and loss of retinoblastoma (RB) function. Restoration of SMURF2 levels in breast cancer cells inhibits cell proliferation and invasiveness, highlighting its therapeutic potential (19). In clear cell renal cell carcinoma, SMURF2 overexpression is associated with improved disease-free and overall survival rates, suggesting its prognostic value. This role of SMURF2 as a tumor suppressor aligns with its involvement in cellular senescence and stability of tumor-suppressing pathways (20, 21).

HIF1α mediates the cellular response to hypoxia, which is a hallmark of the tumor microenvironment in many solid tumors. Under hypoxic conditions, HIF1α stabilizes and translocate to the nucleus, where it activates transcription of genes involved in glycolysis, angiogenesis, and survival pathways that support tumor growth and resilience. HIF1α-driven changes promote cellular adaptation, contributing to increased tumor aggression and resistance to therapy (22).

One of SMURF2’s emerging roles is its regulation of HIF1α. As an E3 ubiquitin ligase, SMURF2 promotes the ubiquitination and subsequent degradation of HIF1α, providing a hypoxia-independent pathway for HIF1α regulation. This SMURF2-mediated degradation mechanism bypasses the traditional VHL (von Hippel-Lindau) pathway, offering an alternative strategy to control HIF1α levels in cancers where VHL is mutated, such as in renal cell carcinoma (23). By targeting SMURF2 to modulate HIF1α stability, it may be possible to influence hypoxia-related cancer pathways without directly inhibiting HIF1α itself, presenting a novel therapeutic approach (24).

The interplay between SMURF2 and HIF1α represents a promising avenue for cancer therapy. SMURF2’s role as both a tumor suppressor and an oncogene highlight its complexity and potential as a context-dependent target, while its regulation of HIF1α provides new insights into controlling hypoxia-driven cancer progression (25). Targeting the SMURF2-HIF1α axis could be especially beneficial in tumors with high hypoxia-inducible factor activity or where traditional therapies face resistance. Further research into these mechanisms may enable the development of targeted therapies that exploit this axis, offering hope for improved outcomes in challenging cancers.

## 3 The interplay of cyclin-dependent kinases, HIF1α, and SMURF2

**Figure 1:**
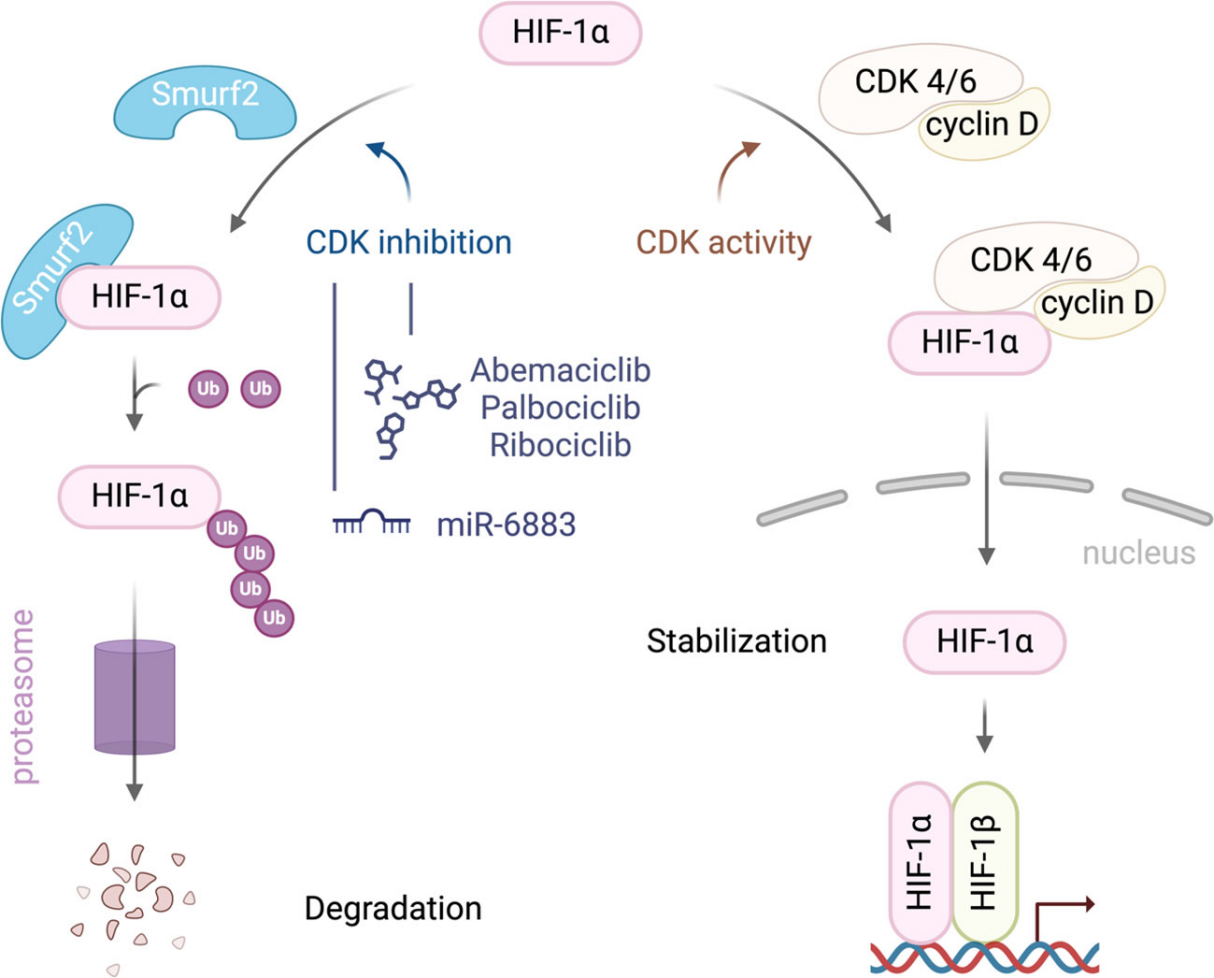
A schematic illustration of the interplay between SMURF2, CDK4/6, and HIF-1α in regulating HIF-1α stability in cancer cells. CDK4/6 activity stabilizes HIF-1α, promoting its accumulation, nuclear translocation, and activation of hypoxia-responsive genes. Inhibiting CDK4/6 enhances the interaction between HIF-1α and the E3 ubiquitin ligase SMURF2 leading to HIF-1α ubiquitination and degradation.

Recent studies have highlighted the complex interactions between CDK4/6, HIF1α, and SMURF2, particularly in regulating HIF1α stability, which has significant implications for cancer progression. SMURF2, an E3 ubiquitin ligase, plays a crucial role in HIF1α degradation, limiting HIF1α’s ability to promote tumor adaptation to hypoxic environments. Research has shown that inhibition of CDK4/6 can activate SMURF2, which in turn enhances the ubiquitination and subsequent proteasomal degradation of HIF1α (**Figure 1**). Notably, this degradation process operates independently of the von Hippel-Lindau (VHL) protein, providing an alternative pathway for controlling HIF1α levels in tumor cells (13).

The therapeutic implications of this interaction are profound. By reducing HIF1α levels, SMURF2-mediated degradation disrupts key adaptive responses in tumors, such as angiogenesis and metabolic reprogramming, both essential for cancer cell survival in hypoxic conditions. Thus, targeting this pathway could inhibit tumor growth and progression, suggesting that therapies combining CDK4/6 inhibitors with agents that modulate SMURF2 activity could enhance anti-cancer efficacy (13).

Further, studies demonstrate that CDK4/6 inhibitors, such as palbociclib, synergize with HSP90 inhibitors to reduce HIF1α levels and suppress cancer cell viability, even in Rb-deficient tumors. This dual-targeting approach leverages complementary HIF1α degradation pathways, underscoring the therapeutic potential of modulating SMURF2 alongside CDK4/6 inhibition. Research by Fassl et al. (26) emphasizes that CDK4/6 inhibition impacts not only cell cycle arrest but also disrupts HIF1α stabilization and downstream hypoxic responses. The reduction in angiogenesis and alteration of tumor metabolism limits cancer cell adaptation to hypoxia (26).

Additionally, recent work by Jensen-Velez et al. (27) highlights the role of miR-6883 in downregulating HIF1α in colorectal and breast cancer cells. Their findings underscore the potential of targeting HIF1α pathways, including through SMURF2 modulation, to impede tumor progression. This insight into miRNA involvement complements the therapeutic strategies that incorporate CDK4/6 inhibition, as reduced HIF1α levels further sensitizes tumors to other anti-cancer agents (27).

Moreover, Zhang et al. (28) report that under hypoxic conditions, CDK4/6 inhibition with agents like palbociclib not only blocks cell proliferation but also reduces HIF1α accumulation, enhancing cancer cell sensitivity to apoptotic signals. This action is particularly effective when combined with chemotherapeutic agents like irinotecan, which promotes colorectal cancer cell death under hypoxia. Together, these findings suggest that CDK4/6 inhibitors may synergize with SMURF2’s role in degrading HIF1α, presenting a promising combination strategy to target hypoxic pathways in cancer therapy (28).

More specifically, targeting CDK1 has shown efficacy in disrupting these pathways by inducing phosphorylation of HIF-1α or through lysosome-inducing degradation of HIF-1α, leading to reduced tumor growth and viability (29). However, targeting CDK1 also presents challenges, such as significant off-target effects and the development of resistance. This is particularly relevant when considering dual inhibition strategies involving CDK1 and HIF1 pathways. For instance, leveraging CDK1’s role in the degradation of HIF1α could fine-tune therapeutic approaches aimed at destabilizing HIF1α under hypoxic conditions, potentially enhancing the efficacy of SMURF2-based therapies, which typically involve the VHL pathway for the ubiquitination and degradation of HIF1α (28, 30).

Separately, studies on CDK4/6 inhibitors like palbociclib suggest that these inhibitors can reduce HIF-1α levels through an indirect mechanism, offering a novel approach to destabilizing HIF1α in a VHL-independent manner and potentially enhancing therapeutic outcomes (31). Recent studies also demonstrate the efficacy of combining CDK4/6 with HSP-90 inhibitors, further inhibiting HIF1 activity and offering potential treatment enhancements for renal and colon cancers (26). This synthesis of findings highlights the potential of kinase-targeted strategies and the importance of continued research into CDK inhibitors to combat tumors characterized by active HIF1α.

## 4 The role of HIF1α in the TME

HIF1α enables cancer cells to thrive in the hypoxic conditions of the TME by regulating genes essential for survival, proliferation, and metabolic adaptation. Recent findings indicate that SMURF2, through its E3 ubiquitin ligase activity, targets HIF1α for degradation, disrupting its ability to drive processes like angiogenesis, metabolic reprogramming, and invasion. This suggests that enhancing SMURF2 activity could be a viable strategy to mitigate the effects of HIF1α-driven tumor progression, particularly in hypoxia-adapted tumors.

### 4.1 HIF1 and the Warburg effect

The Warburg effect, originally described by Otto Warburg in the 1920s, highlights the metabolic reprogramming of cancer cells, which prefer glycolysis over oxidative phosphorylation even in the presence of oxygen. This shift, regulated by the transcription factor HIF1, supports rapid tumor proliferation by enhancing glycolytic flux and creating an acidic TME that promotes malignancy and resistance to therapy. Additionally, this metabolic alteration boosts glutamine metabolism and redirects glycolytic intermediates into biosynthetic pathways essential for tumor growth. Understanding HIF1’s role in driving this metabolic plasticity is crucial for developing novel therapeutic strategies targeting cancer metabolism (4, 6, 14, 32–34). Given SMURF2’s role in protein ubiquitination, it may intersect with these pathways by influencing HIF1 stability and activity, thus impacting the Warburg effect and its contributions to tumor growth. Investigating the regulatory relationship between SMURF2 and HIF1 could provide new insights into targeting the metabolic vulnerabilities of cancer cells.

## 5 Impact on tumor progression and metastasis

HIF1α’s influence extends beyond oxygen adaptation, playing a crucial role in tumor growth and metastasis by upregulating factors that promote cell division and inhibit apoptosis. This promotes tumor invasiveness through the EMT, thereby facilitating metastasis. Hypoxia-induced mitochondrial reactive oxygen species (mROS) further stabilize HIF1α by activating NF-κB via c-SRC-mediated phosphorylation of IκB-α, which is essential for cell viability under hypoxia conditions (35, 36). However, the role of mROS in regulating HIF1α has been debated, with studies presenting conflicting evidence. Recent research suggests that HIF prolyl hydroxylases may not be physiological targets of ROS, and the increase in ROS due to Thioredoxin Reductase downregulation in hypoxia might not significantly affect HIF1α stabilization. Thus, the role of ROS in HIF1α regulation remains an ongoing area of investigation (37–39).

In this context, SMURF2 may intersect with these processes by influencing the ubiquitination and degradation of key proteins involved in the HIF1α signaling pathway. SMURF2’s regulation of protein stability could impact HIF1α activity and, consequently, its role in promoting tumor progression and metastasis. Investigating SMURF2’s interactions within this pathway could provide valuable insights into the mechanisms driving tumor invasion and metastasis, offering potential targets for therapeutic intervention.

### 5.1 Regulatory role in angiogenesis and metabolic reprogramming

Hypoxia-inducible factor 1-alpha (HIF1α) is a crucial regulator of cellular adaptation to low oxygen conditions, significantly influencing tumor progression through the promotion of angiogenesis and metabolic reprogramming. Under hypoxic conditions, HIF1α upregulates pro-angiogenic factors, notably vascular endothelial growth factor (VEGF), which stimulates the formation of new blood vessels. This angiogenesis enhances the supply of nutrients and oxygen to growing tumors, supporting their expansion and survival. The activation of genes such as VEGFA and VEGFR1 under hypoxic conditions underscores the potential of HIF1α and VEGF as biomarkers for aggressive cancer phenotypes, indicating tumor aggressiveness and metastatic potential, which is valuable for prognosis and treatment planning (25, 40–42).

In the complex tumor microenvironment (TME), HIF1α’s upregulation of VEGF and other pro-angiogenic factors not only facilitates tumor growth and invasion but also contributes to immune suppression. Angiogenesis driven by tumor-associated leukocytes and stromal cells within the TME ensures an adequate nutrient and oxygen supply, thereby aiding tumor resilience and resistance to therapy (25, 43, 44). Given HIF1α’s central role in these processes, it presents a promising target for therapies aimed at disrupting angiogenesis and metabolic reprogramming to slow tumor progression and enhance treatment efficacy (43, 45).

SMURF2, an E3 ubiquitin ligase, intersects with HIF1α pathways by modulating the stability and degradation of proteins involved in HIF1α signaling, particularly those associated with VEGF expression and angiogenesis. By regulating the ubiquitination of key factors in the HIF1α pathway, SMURF2 influences the angiogenic and metabolic dynamics within the TME. This interaction suggests that SMURF2 could play a role in destabilizing HIF1α-related signaling, thereby indirectly affecting the tumor’s adaptation to hypoxic conditions. Exploring SMURF2’s role in this context could reveal novel strategies for targeting tumor angiogenesis and improving therapeutic outcomes (41, 46).

Given the central role of HIF1α in tumor adaptation to hypoxia, targeting this pathway offers a strategic approach to disrupt essential processes in tumor progression. Therapies that inhibit HIF1α directly or modulate its associated regulatory mechanisms, such as the SMURF2 pathway, have the potential to impair tumor angiogenesis, reduce immune suppression, and reprogram tumor metabolism. This approach may not only slow down tumor growth but also enhance the effectiveness of existing treatments by sensitizing tumors to therapies that were previously ineffective in the hypoxic TME. Additionally, targeting SMURF2-mediated ubiquitination within the HIF1α pathway could yield combinatorial benefits, presenting a dual mechanism to impair both HIF1α stability and angiogenic signaling (47).

In conclusion, HIF1α is a central regulator of tumor adaptation to hypoxic conditions through its influence on angiogenesis and metabolic reprogramming, which support tumor growth and survival. The critical role of HIF1α and VEGF as biomarkers for aggressive cancer phenotypes highlights the therapeutic importance of targeting these pathways. SMURF2’s role in modulating HIF1α stability further underscores its potential as a therapeutic target in cancer. By focusing on the HIF1α-SMURF2 axis, researchers and clinicians could develop innovative strategies to combat tumor progression, improve treatment responses, and potentially overcome resistance in cancer therapy (48, 49).

## 6 HIF1α’s impact on cancer metabolism and invasion

HIF1α, recognized as an oncogene, is central to the adaptation of cancer cells to the hostile TME. Its role extends beyond simple metabolic transformation via the Warburg effect, involving the enhancement of aerobic glycolysis for rapid ATP production, acidification of the microenvironment, promotion of tumor progression, and increased therapeutic resistance. HIF1α also controls the integration of the crucial energy substrate glutamine into the TCA cycle and lipid biosynthesis, thus fostering anabolic growth (5–7, 33, 50).

In this intricate biological framework, the interaction between HIF1α and SMURF2 emerges as pivotal. SMURF2, through its regulatory role in protein ubiquitination, potentially influences HIF1α stability and activity. The SMURF2-mediated degradation of HIF1α could present a therapeutic avenue to disrupt cancer cells’ adaptive mechanisms, thereby mitigating treatment resistance (13).

Moreover, the HIF1α pathway is crucial in epithelial tissue development and significantly influences oncogenic invasion. It degrades the extracellular matrix (ECM), facilitates epithelial-mesenchymal transition (EMT), and enhances cell motility and invasiveness (9, 51, 52). The activation of this pathway in cancer contributes further to tumor invasiveness and metastasis. The cytokine transforming growth factor-β (TGFβ) is also critical in inducing EMT. Recent research by Chandhoke and colleagues has demonstrated that SMURF2, through sumoylation-regulated mechanisms, effectively suppresses TGFβ-induced EMT (53). This finding highlights the broader significance of the SMURF2-HIF1α axis in cancer biology.

Furthermore, HIF1α promotes cancer invasion, particularly in aggressive cancers like breast cancer, by elevating matrix metalloproteinases (MMPs) and EMT markers. MMPs break down the extracellular matrix, aiding cancer invasion, while EMT markers are commonly associated with aggressive cancers, enhancing survival and growth in low-oxygen conditions (9, 51, 52, 54). This function of HIF1α is pivotal to its role in oncogenesis (55). The critical interplay within the SMURF2-HIF1α axis highlights its potential as a scientific foundation for developing therapeutic strategies aimed at halting cancer progression and improving clinical outcomes. By targeting this axis, it may be possible to disrupt key processes that drive tumor growth and resistance to treatment.

## 7 Mechanisms of HIF1α-induced therapeutic resistance and intervention

HIF1α is a key player in various mechanisms of therapeutic resistance, including enhanced DNA repair, immune evasion, and resistance to apoptosis. These functions are significantly influenced by the SMURF2-HIF1α interaction. SMURF2 promotes the degradation of HIF1α, thereby reducing the hypoxic adaptation mechanisms that confer resistance to treatments like chemotherapy and radiotherapy. This presents an opportunity to develop therapies that enhance SMURF2 activity, potentially overcoming resistance and improving treatment efficacy in hypoxic tumors.

HIF1α is crucial in mediating resistance to radiation and certain chemotherapy treatments (24). Notably, HIF1α activates the expression of genes such as MDR1/P-glycoprotein (P-gp), a key efflux pump involved in multidrug resistance, which expels chemotherapeutic drugs from cancer cells, reducing their intracellular levels and effectiveness (56). For example, advanced colon cancers expressing both HIF1α and P-gp exhibit higher resistance to chemotherapy (57). Inhibiting HIF1α can reverse multidrug resistance by reducing MDR1/P-glycoprotein levels (56). Additionally, HIF1α enhances resistance to radiotherapy in solid tumors by promoting endothelial cell proliferation post-treatment and upregulating genes critical for radio-resistance, such as VEGF, CA9, and GLUT1 (58).

HIF1α also promotes cancer cell survival and therapy resistance by inhibiting apoptosis and encouraging autophagy through the upregulation of genes that prevent apoptosis and support autophagy under treatment stress (59). Other mechanisms of HIF1α’s role in chemo- and radio-resistance include activating DNA repair pathways, which allow cancer cells to survive despite treatment-induced DNA damage (60). This is evident in cancers like glioblastoma, hepatocarcinoma, and lung cancer, where HIF1α enhances DNA repair, contributing to resistance against therapies (60).

SMURF2’s role extends beyond maintaining protein homeostasis, encompassing its critical involvement in the ubiquitination and degradation of proteins involved in cell cycle regulation, DNA damage response, and carcinogenesis through its E3 ubiquitin ligase activity (13, 61–64). **Table 1** presents an overview of the various mechanisms through which HIF1α contributes to therapeutic resistance in cancer and points out about the importance of targeting these pathways to enhance treatment efficacy (65, 66, 68, 69). Cancer’s adeptness at evading therapeutic interventions poses a significant hurdle to effective These functions emphasize the necessity for therapeutic approaches that can circumvent or neutralize HIF1α’s protective effects (1, 13, 70, 71).

**Table 1:** HIF1α-Induced Mechanisms Contributing to Therapeutic Resistance in Cancer.

Mechanism	Description	References
Metabolic Reprogramming	Enhances glycolysis and glutamine metabolism, adapting to hypoxia and aiding cell survival (Warburg Effect).	(4, 14)
Angiogenesis	Promotes blood vessel formation through VEGF upregulation, increasing nutrient/oxygen supply.	(65)
Epithelial-Mesenchymal Transition	Enhances tumor invasiveness and metastatic potential through EMT promotion.	(66)
DNA Repair and Cell Cycle Regulation	Influences genes involved in DNA repair and cell cycle, aiding survival despite treatment.	(67)
Immune Evasion	Leads to suppressed immune surveillance by modulating the TME	(67)
Resistance to Cell Death	Activates resistance against programmed cell death pathways, including apoptosis and ferroptosis.	(1)
Multidrug Resistance (MDR1/P-glycoprotein)	Enhances expression of MDR1/P-glycoprotein, increasing drug efflux and reducing chemotherapeutic efficacy.	(56)
Radio-resistance Enhancement	Upregulates genes like VEGF, CA9, and GLUT1, crucial for enhancing resistance to radiotherapy.	(58)
Autophagy Promotion	Promotes autophagy under treatment stress, enhancing cell survival by inhibiting apoptosis.	(59)

Of particular interest, SMURF2’s role extends beyond maintaining protein homeostasis, encompassing its crucial involvement in the ubiquitination and subsequent degradation of proteins involved in cell cycle regulation, DNA damage response, and carcinogenesis through its E3 ubiquitin ligase activity. By modulating protein degradation pathways, the SMURF2-HIF1α pathway profoundly impacts the stability and function of proteins pivotal for the effectiveness of therapies targeting key oncogenic drivers like EGFR, KRAS, BRAF, and c-Myc (72–74). Cancer cells exploit this dysregulated protein degradation, mediated through the SMURF2-HIF1α pathway, to diminish cellular levels of proteins essential for the accurate repair of DNA damage or maintaining signal transduction fidelity, leading to an increased capacity for DNA repair and altered responsiveness to mutation-targeted therapies. Targeting the SMURF2-HIF1α axis to modulate the degradation of critical proteins involved in therapeutic resistance could enhance cancer cells’ sensitivity to targeted therapies, offering a promising strategy to counteract these resistance mechanisms. This approach, combined with the potential of combination therapies involving CDK and HSP-90 inhibitors, could significantly impact cancer progression and drug resistance by stabilizing HIF1α, suggesting a synergistic mechanism that broadens the scope of combating therapy resistance (1, 75).

By focusing on the intersection between the SMURF2-HIF1α pathway and protein degradation, new avenues can be unlocked to combat the ever-evolving landscape of therapeutic resistance in cancer. This will pave the way for novel therapeutic strategies that more effectively disrupt the adaptive mechanisms cancer cells employ to evade treatment.

## 8 Heat shock proteins in cancer: modulating the SMURF2-HIF1α axis to overcome resistance

Combining cyclin-dependent kinase (CDK) inhibitors with heat shock protein 90 (HSP-90) inhibitors presents a promising approach to destabilizing hypoxia-inducible factor 1-alpha (HIF1α) and inhibiting cancer cell growth. HIF1α, a transcription factor essential for tumor adaptation under hypoxic conditions, drives pathways involved in angiogenesis, metabolism, and cellular survival. CDK1 has been shown to stabilize HIF1α via phosphorylation at Ser668, a process independent of the von Hippel-Lindau (VHL) protein pathway, which is crucial for maintaining HIF1α stability. Inhibiting CDK1 disrupts this phosphorylation, leading to decreased HIF1α stability and impairing cancer cell survival mechanisms (31). Furthermore, HSP-90, a molecular chaperone, ensures the proper folding and function of various oncogenic proteins, including HIF1α. HSP-90 inhibition induces the misfolding and proteasomal degradation of its client proteins, reducing HIF1α levels and diminishing tumor adaptive responses to hypoxia (76, 77).

The study of heat shock proteins (HSPs) in cancer has further highlighted their roles in progression and therapy resistance, particularly the critical role of HSP-90 in stabilizing oncogenic proteins. HSP-90 emerges as a significant anticancer target (78, 79); however, compensatory induction of HSP-70 following HSP-90 inhibition reduces the efficacy of HSP-90 inhibitors by counteracting their therapeutic impact (79, 80). This compensatory mechanism is evident in the dynamic interplay between the SMURF2-HIF1α pathway and HSPs, where HSP-70 influences cellular responses to hypoxia and affects lung cancer recurrence, particularly following treatments such as radiofrequency ablation (81). For instance, HSP-70’s interaction with HIF1α in non-small cell lung cancer (NSCLC) has been shown to impede ferroptosis, a regulated cell death process that could otherwise counteract therapy resistance (77, 82, 83). This distinction highlights HSP-70’s unique role in cancer biology, where its inhibition of ferroptosis and promotion of therapy resistance contribute to cancer persistence, marking it as an essential factor in strategies to target HSP pathways.

The dual inhibition of CDKs and HSP-90 amplifies the reduction of HIF1α levels compared to either inhibitor alone, as this approach disrupts both phosphorylation-dependent stabilization and chaperone-mediated folding of HIF1α. Such synergy offers an effective strategy to impair cancer cell growth by targeting HIF1α stability through complementary mechanisms (84, 85). However, recent studies reveal challenges in HSP-targeted strategies, as Phase I trials combining HSP-90 inhibitor ganetespib with ziv-aflibercept encountered significant toxicity issues while aiming to counter resistance by concurrently targeting HIF1α stabilization and angiogenesis (86).

Research into strategies such as the concurrent inhibition of all HSP-90 paralogs and disruption of HSP90-Cdc37 protein interactions seeks to enhance the anticancer efficacy of HSP-90 inhibitors and mitigate compensatory effects from HSP-70 (87). This intricate interplay between HSP-90, HSP-70, and the SMURF2-HIF1α axis underscores the necessity of carefully modulated therapies that target these pathways, aiming to overcome cancer cells’ adaptive mechanisms to achieve more durable responses. Together, these insights into CDK and HSP inhibition underscore the potential of leveraging cellular mechanisms to combat cancer, offering promising avenues for improved and lasting therapeutic strategies in oncology (86).

## 9 The role of SMURF2 in maintaining genome integrity and protein homeostasis

The role of SMURF2 extends to maintaining genomic integrity, targeting specific proteins for degradation to prevent aneuploidy and genomic instability—key hallmarks of cancer (1, 75). One mechanism is through TGF-β/Bone Morphogenetic Protein (BMP) signaling—commonly referred to as the TGF-β/BMP pathway—along with its role in maintaining protein homeostasis and genomic integrity, positions it as a critical target for innovative cancer treatment strategies (3, 88, 89).

TGF-β, bone morphogenetic proteins (BMPs), and activins—proteins that help regulate various biological functions, including cell growth and differentiation— play crucial roles in the TME by regulating cellular processes such as proliferation, migration, and invasion through EMT. These signaling molecules also modify stromal phenotypes, impacting extracellular matrix composition, angiogenesis, immune modulation, and inflammation. Given their profound influence on tumor behavior and microenvironment, TGF-β, BMP, and activin pathways are promising targets for developing new cancer therapies to control or reverse tumor progression (90).

SMURF2 is essential for maintaining cellular protein homeostasis and managing stress through its involvement in the Ubiquitin-Proteasome System (UPS), which removes damaged or surplus proteins. It also plays a role in regulating cell growth by overseeing protein degradation during cancer progression. Additionally, the Autophagy-Lysosome Pathway (ALP) helps break down larger cellular structures in response to stress conditions such as nutrient deficiencies. UPS and ALP are pivotal in cancer cell metabolism and maintaining protein equilibrium, which is crucial for supporting energy production and cell division. Disruptions in the HIF-1α/SMURF2 axis can impair these processes, providing potential targets for improving cancer treatments and overcoming resistance to existing therapies (91, 92). Additionally, recent studies have unveiled SMURF2’s pivotal role in modulating KAP1, a critical nuclear factor involved in transcriptional repression and maintaining genomic integrity. Shah and colleagues (46) demonstrated that SMURF2 substantially affects KAP1’s protein-protein interactions, influencing key cellular functions like proliferation and DNA damage response. This expanded understanding of SMURF2’s regulatory capabilities further highlights its therapeutic potential in targeting complex networks within cancer cells (46). Furthermore, SMURF2 plays a pivotal role in genomic integrity maintenance by targeting specific proteins involved in the cell cycle and DNA repair mechanisms for degradation. This activity ensures proper chromosomal segregation during cell division, preventing aneuploidy and genomic instability, hallmarks of cancer cells, thereby reinforcing SMURF2’s function as a tumor suppressor (1, 75).

SMURF2 undergoes posttranslational modifications, including phosphorylation and ubiquitination, which affect its stability, activity, and interactions. Additionally, SMURF2 modulates the stability and function of proteins like KAP1 in cancer cells and targets TGF-β receptors for degradation, impacting cancer cell behavior and TGF-β signaling (46, 93). Thus, SMURF2 emerges as a cornerstone in cancer therapeutics. Its multifaceted role in modulating genomic stability and protein homeostasis offers a promising avenue for developing targeted treatments that could profoundly improve cancer therapy outcomes.

## 10 Clinical development of HIF1α inhibitors

### 10.1 Emerging therapeutics targeting HIF1α

**Figure 2:**
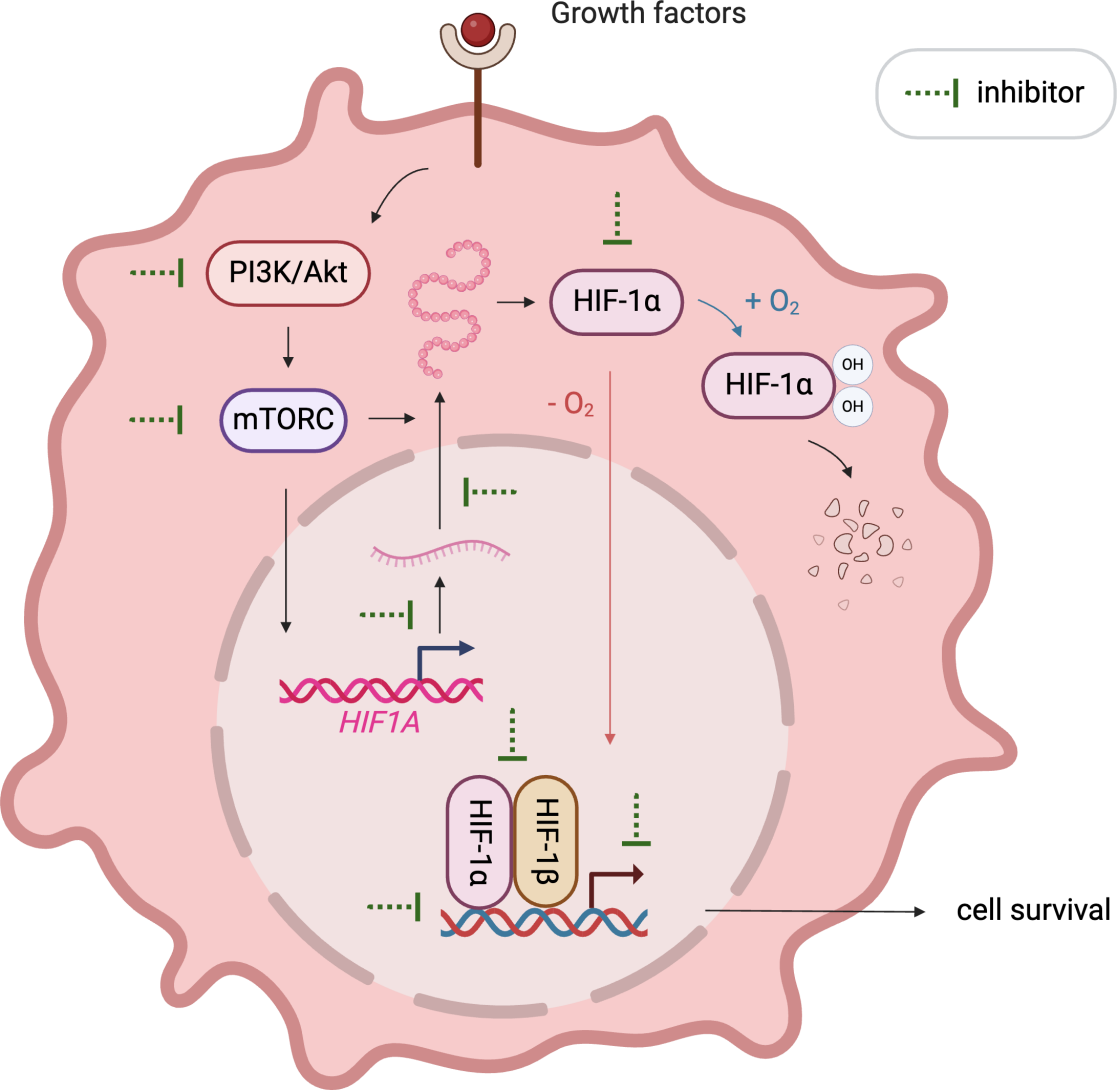
Illustration of emerging approaches targeting HIF1α. Various therapeutics inhibit HIF1α signaling by affecting its mRNA expression, protein synthesis, stabilization, dimerization with HIF1β, DNA binding, and transcriptional activity.

The crucial role of HIF1α in cancer progression has catalyzed the development of numerous inhibitors targeting its various functions, which are currently under investigation in pre-clinical and clinical stages. These inhibitors disrupt HIF1α’s mRNA expression, protein synthesis, stabilization, dimerization with HIF1β, DNA binding, and transcriptional activity (**Figure 2**). Notably, MO-460, a compound derived from moracin-O, impedes HIF1α accumulation by targeting the protein heterogeneous nuclear ribonucleoprotein A2/B1 (hnRNPA2B1), inhibiting the initiation of HIF1α translation—a novel mechanism of action (94). Additionally, by disrupting HIF1α’s transcriptional activities, acriflavine has shown efficacy in melanoma cells by impairing pathways crucial for cancer cell survival, including angiogenesis and glycolysis (95, 96).

### 10.2 Clinical trials and studies

Various clinical studies further assess the efficacy of the inhibitors targeting the HIF pathway (**Table 2**). A study registered under ClinicalTrials.gov ID NCT01763931 investigated the pharmacodynamics of IG-HIF1 in breast cancer, revealing insights into HIF1α’s role in predicting tamoxifen resistance (97). Another trial (NCT05119335) examines NKT2152, a HIF2α inhibitor, in advanced (ccRCC), exploring its potential to disrupt cancer growth by inhibiting both HIF2α and CDK4/6 (98).

**Table 2:** Clinical Trials targeting the HIF pathway.

Inhibitor Name	Mechanism of Action	Targeted Pathway/Function	Clinical Trial ID
IG-HIF1	Predicts tamoxifen resistance via HIF1α	HIF1α in breast cancer	NCT01763931
NKT2152	Inhibits HIF2α and CDK4/6	HIF2α	NCT05119335
CRLX101 + Bevacizumab	Combination therapy targeting HIF1α	HIF1α in recurrent cancers	NCT01652079
PX-478	Reduces HIF1α protein levels	HIF1α stabilization	NCT00522652

Additionally, combination therapy involving CRLX101 with Bevacizumab is under investigation for recurrent ovarian, tubal, and peritoneal cancer (NCT01652079), indicating the broadening scope of HIF1α targeted therapies. Additionally, research from the National Cancer Institute indicates that sustained inhibition of Topoisomerase I (Topo I) may activate a novel HIF1α inhibition mechanism that is Topo I-dependent (99).

Moreover, inhibitors like PX-478, EZN-2968 and other small molecules targeting HIF1α effectively reduce HIF1α protein levels, contributing to tumor regression and, in some cases, leading to prolonged disease stabilization (100–102).

Thus, the crucial role of HIF1α in cancer progression has catalyzed the development of numerous inhibitors targeting its various functions, which are currently under investigation in preclinical and clinical stages. These inhibitors disrupt HIF1α’s mRNA expression, protein synthesis, stabilization, dimerization with HIF1β, DNA binding, and transcriptional activity. In light of the emerging importance of the SMURF2-HIF1α axis, these developments could be enhanced by combining HIF1α inhibitors with strategies that modulate SMURF2 activity. Such combination therapies may improve outcomes in cancers where SMURF2-mediated degradation of HIF1α is a key factor in therapeutic success. A comprehensive illustration in this section depicts these therapeutic strategies and ongoing clinical trials, emphasizing the integrated approach needed to target HIF1α effectively.

## 11 Strategic targeting of the SMURF2-HIF1α axis in cancer therapy

Targeting the SMURF2-HIF1α axis represents a novel strategy to counteract cancer adaptations within TME. This approach capitalizes on orchestrating the ubiquitin-proteasome mediated degradation of HIF1, a pivotal regulator of cellular responses to hypoxia. SMURF2 recognizes HIF1 through defined regions or motifs, including specialized amino acid sequences, hydrophobic patches, or structural conformations altered by oxygen levels or other signaling molecules. Once bound, SMURF2 acts as an E3 ubiquitin ligase, catalyzing the transfer of ubiquitin to lysine residues on HIF1, initiating its polyubiquitination. This process marks HIF1 for recognition by the proteasome, which degrades tagged proteins into peptides for recycling. Such degradation is crucial for modulating HIF1 activity under varying cellular conditions, thereby maintaining tight control over hypoxia-inducible transcription factors that influence gene expression related to angiogenesis, metabolism, and tumor progression, potentially increasing tumor susceptibility to immune-mediated destruction and improving responsiveness to traditional therapies. Furthermore, specific phosphorylation sites and protein-protein interaction interfaces on HIF1 may influence SMURF2’s recognition and ubiquitination efficiency, suggesting a layer of regulatory complexity that could be exploited therapeutically. Disruption of these molecular interactions or modification of key degron sequences within HIF1 may alter its stability, offering potential targets for drug development to modulate HIF1 levels in hypoxia-driven pathologies such as cancer. Exploring the SMURF2-HIF1α pathway within the TME facilitates the development of targeted treatments that circumvent traditional resistance mechanisms and enhance patient outcomes. However, targeting this pathway involves challenges such as achieving intervention specificity and managing potential unintended cellular impacts, given SMURF2’s dual roles in regulating genomic stability and protein homeostasis. Despite these challenges, manipulating this pathway could revolutionize cancer therapy, offering new hope against resistant tumor types (3, 13, 46).

The recent discovery that SMURF2 promotes ferroptosis by degrading GSTP1 introduces new approaches for treating tumors resistant to standard treatments (103). Future research should focus on developing specific SMURF2 inhibitors to enhance cancer cell sensitivity to ferroptosis, potentially overcoming resistance to other cell death mechanisms. Additionally, modulating GSTP1 could protect normal cells or increase cancer cell susceptibility to ferroptosis. Investigating other SMURF2 targets and interactions with GSTP1 within the TME, especially involving immune cells, could further elucidate its role in cancer survival and unveil new immunotherapeutic opportunities.

Leveraging the relationship between HIF1α and the TME through the SMURF2-HIF1α axis offers a multifaceted strategy for cancer treatment, particularly beneficial in tumors resistant to traditional therapies targeting the von Hippel-Lindau-HIF1α pathway (75, 104, 105).

An overview of targeted approaches against HIF1α is essential. **Table 3** delineates a spectrum of inhibitory strategies, each targeting distinct stages of HIF1-alpha’s lifecycle from transcriptional initiation to degradation, showcasing the depth of potential interventions. Among these, enhancing SMURF2 activity is a novel method with significant therapeutic impact, especially in combating tumors exhibiting von Hippel-Lindau dysregulation or persistent hypoxia.

**Table 3:** Overview of HIF1α Modulation Strategies.

Strategy Category	Examples	Mechanism of Action
Transcriptional Initiation Inhibitors	Flavopiridol, Aminoflavone. α-amanitin, Actinomycin D, Triptolide	Target nascent phase of HIF1α
mRNA Stabilization Inhibitors	EZN-2968	Bind HIF1α mRNA, preventing stabilization
Translational Initiation Inhibitors	Rapamycin, Cetuximab, Buparlisib, KC7F2, mTOR inhibitors	Assault translational genesis of HIF1α
Microtubule Dynamics and Na+/K+ ATPase	2-MEs, Digoxin	Indirectly impact HIF1α stability
ATR-Mediated Regulation	AZD6738, VX-970	Modulate HIF1α translation
HSP-90 Stability Inhibitors	Ganetespib, Apigenin, Lonafarnib, 17-AAG, 17-DMAG	Destabilize HIF1α via HSP-90 targeting
HDAC-Mediated Stability Inhibition	Vorinostat (SAHA), Romidepsin (FK228), Trichostatin A, Valproic acid	Modulate HIF1α stability and transcriptional activities
Dimerization and DNA Binding Inhibition	Acriflavine, Echinomycin, Anthracycline	Obstruct HIF-1’s activation of hypoxia-responsive genes
Transactivation Activity Inhibitors	Bortezomib	Curtail HIF1α’s transactivation
Multi-level Lifecycle Inhibitors	PX-478, Camptothecin, CRLX101, Metformin	Inhibit HIF1α across various lifecycle stages

Thus, Targeting the SMURF2-HIF1α axis represents a novel approach to counteracting the adaptive mechanisms cancer cells employ within the TME. SMURF2’s ability to ubiquitinate and degrade HIF1α disrupts hypoxia-induced survival pathways, offering a unique therapeutic target. Enhancing SMURF2 activity could be particularly beneficial in cancers resistant to current treatments, especially those driven by hypoxia. Additionally, recent discoveries linking SMURF2 with the promotion of ferroptosis through GSTP1 degradation suggest further therapeutic potential in overcoming resistance to cell death. Future research should focus on the development of specific SMURF2 modulators to exploit these pathways in cancer therapy.

## 12 Implications and challenges in targeting the SMURF2-HIF1α pathway

TME and Therapeutic Challenges: The TME significantly influences the SMURF2-HIF1α pathway, primarily through HIF1α, creating a critical feedback loop essential for tumor adaptation and survival. The interdependence of these mechanisms indicates the pathway’s potential in developing new therapies, especially for tumors resistant to current treatments. **Table 4** is for selected HIF1 inhibitors demonstrated to prevent chemo/radiotherapy resistance in various cancer or cancer cell lines reviewed by Bui and colleagues. This interdependence highlights the significance of this pathway in creating new therapeutic strategies for cancers, especially those resistant to current treatments (24).

**Table 4:** Selected HIF1 inhibitors that underwent clinical or laboratory testing.

HIF1 Inhibitors (Reference)	Anticancer Therapy	Cancer Type/Cell Line	Mechanism for Prevention of Resistance
LW6 (106)	Mitoxantrone, Doxorubicin	Breast cancer	Drug efflux
LC478 (106)	Docetaxel	Colorectal adenocarcinoma	Drug efflux
EZN-2208 (107)	Topotecan	Breast cancer	DNA damage repair
Chetomin (108)	Radiation	Glioma	Metabolism
PX-478 (109)	Radiation, Gemcitabine	Pancreatic cancer	Apoptosis
Acriflavine (110)	Radiation	Rectal cancer	ETM transition Inhibition
PMX290 (111)	5-Fluorouracil	Colon adenocarcinoma	Apoptosis
Romidepsin (112)	Temozolomide	Glioma	Apoptosis
BIX-01294 (113)	TRAIL	Hepatocellular carcinoma	Apoptosis
Bortezomib (114)	Radiation	ccRCC	Binding inhibition p300
LBH589 (115)	Cisplatin, Bortezomib, Osimertinib	Ovarian cancer, Multiple myeloma, Lung cancer	Apoptosis
Vorinostat (35)	Paclitaxel, Doxorubicin, Bortezomib	Breast cancer, Neuroblastoma, Mesothelioma	Apoptosis
NSC-134754 (116)	Cisplatin, Doxorubicin	Osteosarcoma	Apoptosis
YC-1 (117)	Gefitinib, Cisplatin	Lung cancer, Oral cancer	Drug efflux, Apoptosis
Everolimus (118)	Cisplatin	Gastric cancer	Drug efflux, Apoptosis
Lonafarni (119)	Paclitaxel, Cisplatin	Lung cancer, Melanoma	Drug efflux, Apoptosis
Echinomycin (120)	Hormone	Prostate cancer	Not determined

### 12.1 Safety challenges in targeting the SMURF2-HIF1α pathway

While promising, the strategic targeting of the SMURF2-HIF1 pathway in cancer therapy confronts formidable hurdles due to the intricate roles and broad biological activities of SMURF2 and HIF1α. SMURF2, a tumor suppressor, regulates various cellular processes beyond degrading HIF1α, including the ubiquitin-proteasome system’s stability, essential for normal cell function. Similarly, HIF1α is crucial for physiological responses to hypoxia, such as erythropoiesis and metabolic reprogramming. Targeting these factors may lead to unintended systemic effects, like anemia or metabolic dysregulation, and impair tissue repair. The inherent challenge in directly targeting transcription factors like HIF-1, which lack easily accessible active sites, is further complicated because many compounds that inhibit or activate HIF-1 lack isoform specificity. Consequently, any intervention will affect both isoforms, with the impact varying based on the targeted tissue (32, 121). Most available inhibitors are indirect and can alter mRNA expression or protein degradation, risking further off-target effects and potential resistance as cancer cells may activate compensatory pathways.

Some HIF-1α inhibitors have been reported to affect other signaling pathways, leading to off-target effects. For instance, certain inhibitors may interfere with angiogenesis and normal cellular responses to hypoxia, raising concerns about their specificity and safety profiles (12). Inhibitors of HIF-1α, such as those used in treating renal anemia, have been associated with adverse effects like hypertension and thromboembolic events. These systemic effects underscore the need for careful monitoring and patient selection when employing HIF-1α-targeted therapies (122).

SMURF2 plays a role in protein homeostasis and signaling pathways. Inhibiting SMURF2 may disrupt these processes, potentially leading to unintended cellular consequences. This highlights the importance of understanding the broader implications of SMURF2 inhibition in therapeutic contexts (3). Targeting the SMURF2-HIF1α pathway poses significant safety challenges due to SMURF2’s dual role as both a tumor suppressor and oncogene, depending on context. In some cancers, SMURF2 stabilizes proteins like KRAS and EGFR and activates pathways such as Wnt/β-catenin, contributing to tumor progression and resistance to therapy (123). Additionally, loss of SMURF2 can lead to genomic instability, heightening risks for chromosomal aberrations and potentially fostering secondary malignancies (20, 124).

These complexities point to the necessity for precise targeting strategies and the development of combination therapies to mitigate off-target effects and enhance therapeutic outcomes. Logistically, developing specific inhibitors that can precisely target the SMURF2-HIF1 interaction without affecting other pathways is challenging. The complexity of the protein-protein interaction network in which these molecules operate necessitates highly selective and potent inhibitors, which are challenging to design and optimize. Moreover, the pharmacokinetic properties of these inhibitors—ensuring they are delivered effectively to the tumor site without degradation or unacceptable toxicity—pose additional challenges.

Furthermore, resistance mechanisms may develop rapidly due to the genetic plasticity of tumors, potentially rendering SMURF2-HIF1 targeted therapies ineffective over time. Overcoming this would require combination therapies or next-generation inhibitors to prevent or circumvent resistance mechanisms. Finally, the economic implications of developing such targeted therapies cannot be overlooked. The cost of research and development, coupled with the clinical trials necessary to prove efficacy and safety, makes the venture costly. Without clear pathways to reimbursement and market adoption, even clinically successful treatments may fail to become accessible to patients. These challenges highlight the need for precision in targeting these interactions to minimize off-target effects and systemic toxicity. These findings emphasize the necessity for comprehensive preclinical and clinical evaluations to assess the safety and specificity of therapies targeting SMURF2 and HIF1α. Developing strategies to mitigate systemic and off-target effects will be crucial for the successful implementation of these targeted therapies in cancer treatment.

As described, SMURF2 plays a dual role in maintaining protein homeostasis and genomic integrity, making it a crucial element in cancer cell survival and proliferation under hypoxic conditions typical of solid tumors. Furthermore, the pathway’s engagement with other critical cellular processes like the ubiquitin-proteasome and autophagy-lysosome pathways, essential for protein degradation and cellular cleanup, Emphasizes the importance of a targeted approach that can distinguish between its pathological and physiological roles. Such differentiation is critical to developing therapeutic strategies that harness the pathway’s cancer-promoting aspects without disrupting its normal cellular functions.

### 12.2 Expanding clinical horizons and addressing therapeutic targeting

While our review reveals the critical role of the SMURF2-HIF1α pathway in cancer progression and therapy resistance, it is crucial to consider the potential side effects and economic implications of new therapies. Addressing these issues involves a thorough understanding of the pathway’s modulation effects on normal physiological functions, which is crucial for ensuring the safety and efficacy of clinical applications. This knowledge will be instrumental in transitioning from laboratory findings to viable therapeutic options that significantly improve patient care.

### 12.3 Strategic approach to drug development for SMURF2 inhibition

Recent research has led to the development of SMURF2-targeting peptides and peptidomimetics, disrupting the C2-HECT interaction and stimulating SMURF2’s autoubiquitination and turnover. This regulation impacts cancer cell growth and sensitivity to drugs like etoposide. Introducing compounds such as Pep7 reduces SMURF2’s cellular levels, affecting cancer cell proliferation and augmenting cancer therapy by increasing the cytotoxicity of existing treatments (125). This approach incorporates selected modifications in putative inhibitors to probe and optimize the interaction with the SMURF2-HIF1α complex. Lead compounds would also be optimized for efficacy and to enhance their pharmaceutical properties. The focused effort aims to deliver targeted therapeutic agents that effectively manage cancer. At the El-Deiry laboratory, the synthesized compounds will be evaluated to assess their inhibition of the SMURF2-HIF1α interaction selectively. This inhibition is critical to enhancing treatment effectiveness, especially in tumors adapted to low oxygen that resist standard therapies. It is part of a wider strategy to combine these inhibitors with current treatments, blocking survival pathways often activated in resistant tumors. The main aim is to develop precision medicine, customize treatments to specific tumor traits, and thoroughly evaluate these compounds in clinical trials (75, 126).

## 13 SMURF2 and ferroptosis: implications for cancer therapy

Ferroptosis, a form of cell death driven by iron-dependent lipid peroxidation, offers a novel therapeutic approach for treating cancers that are resistant to conventional therapies. The emerging evidence on the role of SMURF2 in modulating ferroptosis by mediating the degradation of glutathione S-transferase P1 (GSTP1) dismantles a key defense against ferroptosis, thereby predisposing cancer cells to this unique form of cell death (103, 127).

Recent studies suggest that SMURF2 targets GSTP1, an antioxidant enzyme, for ubiquitination and proteasomal degradation. GSTP1 normally detoxifies lipid peroxides, preventing the buildup of lipid species that trigger ferroptosis (127). By reducing GSTP1 levels, SMURF2 disrupts this protective mechanism, sensitizing cancer cells to ferroptotic cell death. This pathway operates independently of Glutathione Peroxidase 4 (GPX4), another primary regulator of ferroptosis, suggesting an alternative method through which SMURF2 can enhance ferroptotic susceptibility in cancer cells (103).

This mechanism highlights the potential of targeting the SMURF2-GSTP1 axis as a novel therapeutic strategy to promote ferroptosis and overcome resistance to cell death, especially in tumors that rely on GSTP1 to maintain redox balance and evade ferroptosis. Leveraging SMURF2 to selectively degrade GSTP1 provides a promising approach for driving ferroptosis in cancer cells resistant to conventional therapies (103).

**Figure 3:**
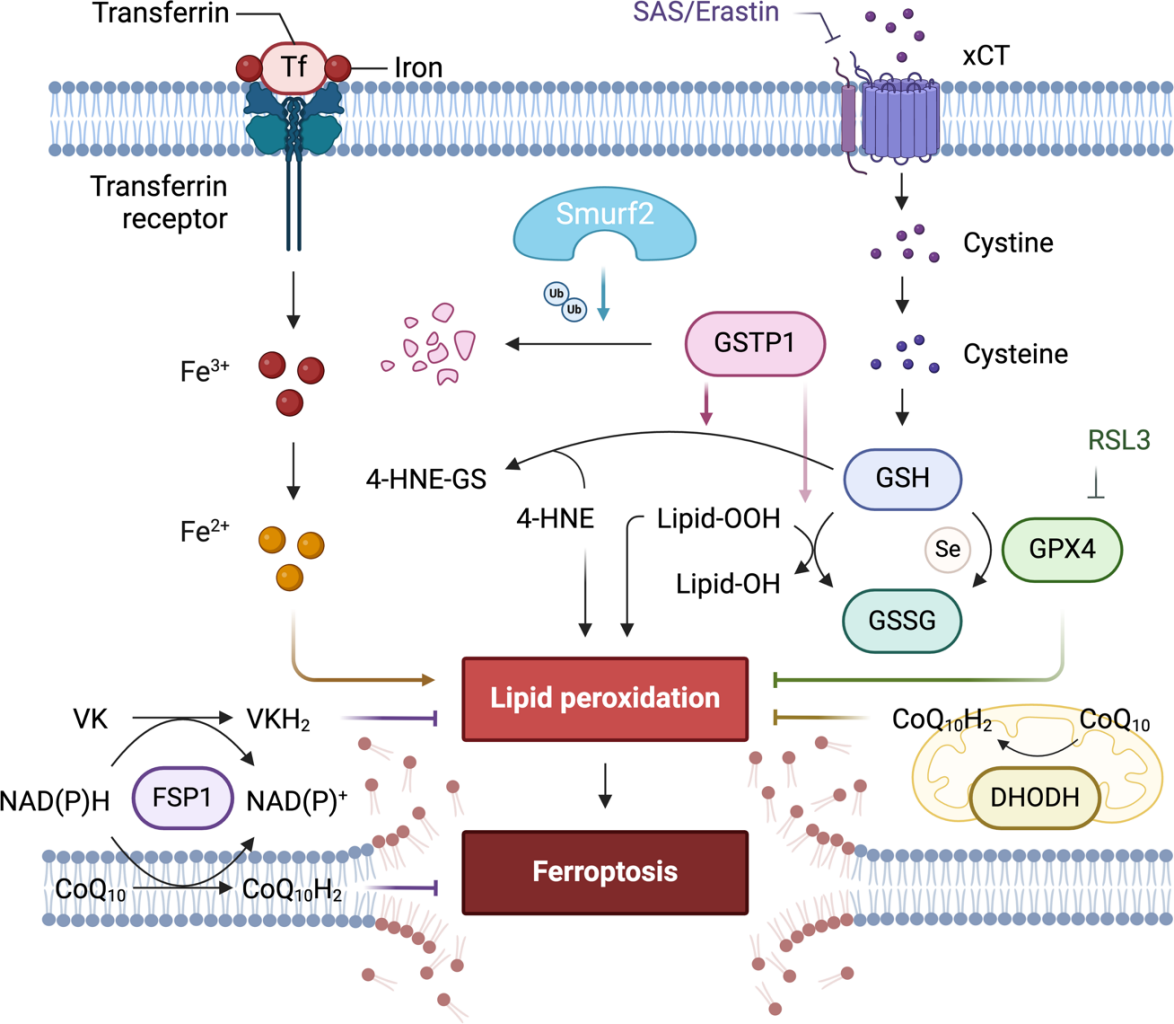
The role of differentially expressed proteins in an early-stage, GPX4-independent ferroptosis regulatory mechanism. It emphasizes the central role of the E3 ligase SMURF2 in managing the degradation of GSTP1. The figure also highlights the significance of GST and selenium independent GPx activities of GSTP1 in safeguarding against ferroptosis. Furthermore, it illustrates how manipulating the SMURF2-GSTP1 equilibrium can heighten the vulnerability of cancer cells to ferroptosis.

The potential of such a novel supporting concept of targeting the SMURF2-GSTP1 interaction to enhance the efficacy of ferroptosis-inducing treatments is particularly significant in cancer types where resistance to apoptosis is high and where therapies targeting other pathways, such as those mediated by GPX4, have shown limited effectiveness (**Figure 3**). The research demonstrated that GSTP1 shields cells from ferroptosis by conjugating glutathione (GSH) with 4-hydroxynonenal (4-HNE). Notably, the role of DHODH in inducing ferroptosis in cancer cells suggests an additional therapeutic angle.

Consequently, overexpression of SMURF2 enhances the sensitivity of tumor cells to ferroptosis induced by sulfasalazine (SAS). This mechanism could be exploited to target cancer cells that have developed resistance mechanisms to traditional therapies (70, 103). Furthermore, the therapeutic manipulation of this pathway could be highly relevant for enhancing the effects of drugs that induce ferroptosis, potentially overcoming the challenges posed by drug-resistant cancer cells. By sensitizing cancer cells to ferroptosis, targeting the SMURF2-GSTP1 axis might offer an additional advantage by circumventing the cellular mechanisms that confer resistance to other forms of cell death (103). Continued research into this pathway promises to expand our arsenal against hard-to-treat cancers. It offers a deeper understanding of how cancer cells evade death and how they might be selectively targeted in a therapeutic context. This strategy reinforces the importance of integrated research efforts that bridge molecular insights with clinical applications, aiming to deliver targeted, effective cancer treatments.

In exploring ferroptosis at its nascent stages, Zhang et al. unveil a regulatory mechanism of ferroptosis that operates independently of GPX4, regulated by the E3 ligase SMURF2 (103). This mechanism centers on SMURF2’s role in the targeted degradation of GSTP1, a critical defender against ferroptosis. Through its enzymatic activity, GSTP1 conjugates GSH with 4-HNE and displays selenium-independent GPx-like activity to detoxify lipid peroxides, thus protecting cells from ferroptotic cell death.

This process highlights its pivotal regulatory function by which SMURF2 mediates cellular responses to oxidative stress by modulating the stability of GSTP1. The degradation of GSTP1 by SMURF2 not only diminishes the cell’s ferroptotic defenses but also sensitizes cancer cells to treatments that induce ferroptosis. This insight into the SMURF2-GSTP1 interaction adds a crucial layer to our understanding of ferroptosis regulation, presenting SMURF2 as a key facilitator of this iron-dependent form of cell death, which could be leveraged to enhance the efficacy of pro-ferroptosis cancer therapies. **Table 5** below presents key research milestones in the study of ferroptosis, providing an essential reference for significant developments in this field, especially highlighting the role of different proteins like SMURF2, Ferroptosis Suppressor Protein 1 (FSP1), and Dihydroorotate Dehydrogenase (DHODH) in regulating this form of cell death Each entry includes a brief focus on the study and the key findings, which collectively emphasize the potential of targeting ferroptosis as a novel approach in cancer therapy.

**Table 5:** Summary of studies that have explored targeting ferroptosis in cancer therapy.

Study/Authors	Focus of Study	Key Findings
Zhang et al. (103)	Role of SMURF2 in ferroptosis	SMURF2 promotes ferroptosis in cancer cells by mediating the degradation of GSTP1.
Doll et al. (128)	FSP1 as a ferroptosis suppressor	FSP1 was identified as a new antioxidant enzyme that functions parallel to GPX4.
Mao et al. (129)	DHODH-regulated ferroptosis	Inhibition of DHODH induces ferroptosis in cancer cells, suggesting a new therapeutic angle.
Bersuker et al. (130)	CoQ oxidoreductase FSP1	FSP1 acts independently of GPX4 to suppress ferroptosis.
Jiang et al. (131)	A comprehensive review of ferroptosis mechanisms and roles	Reviewed the biological roles of ferroptosis and its potential therapeutic applications.

## 14 SMURF2-HIF1α: regulation and potential in cancer therapy

SMURF2 has multiple roles that demonstrate its importance in cancer pathophysiology. SMURF2: A Dual Role in Protein Degradation and Tumor Suppression: Under normoxic conditions, SMURF2 utilizes its E3 ubiquitin ligase activity to ubiquitinate and degrade HIF1α, maintaining low intracellular protein levels. This function shifts under hypoxic tumor conditions, where SMURF2 is crucial for the polyubiquitination and proteasomal degradation of HIF1α. This modulation impacts tumor progression, angiogenesis, metabolic reprogramming, and cell survival, establishing SMURF2 as a potent therapeutic target (13).

### 14.1 SMURF2: a multifaceted regulator in tumor suppression

**Figure 4:**
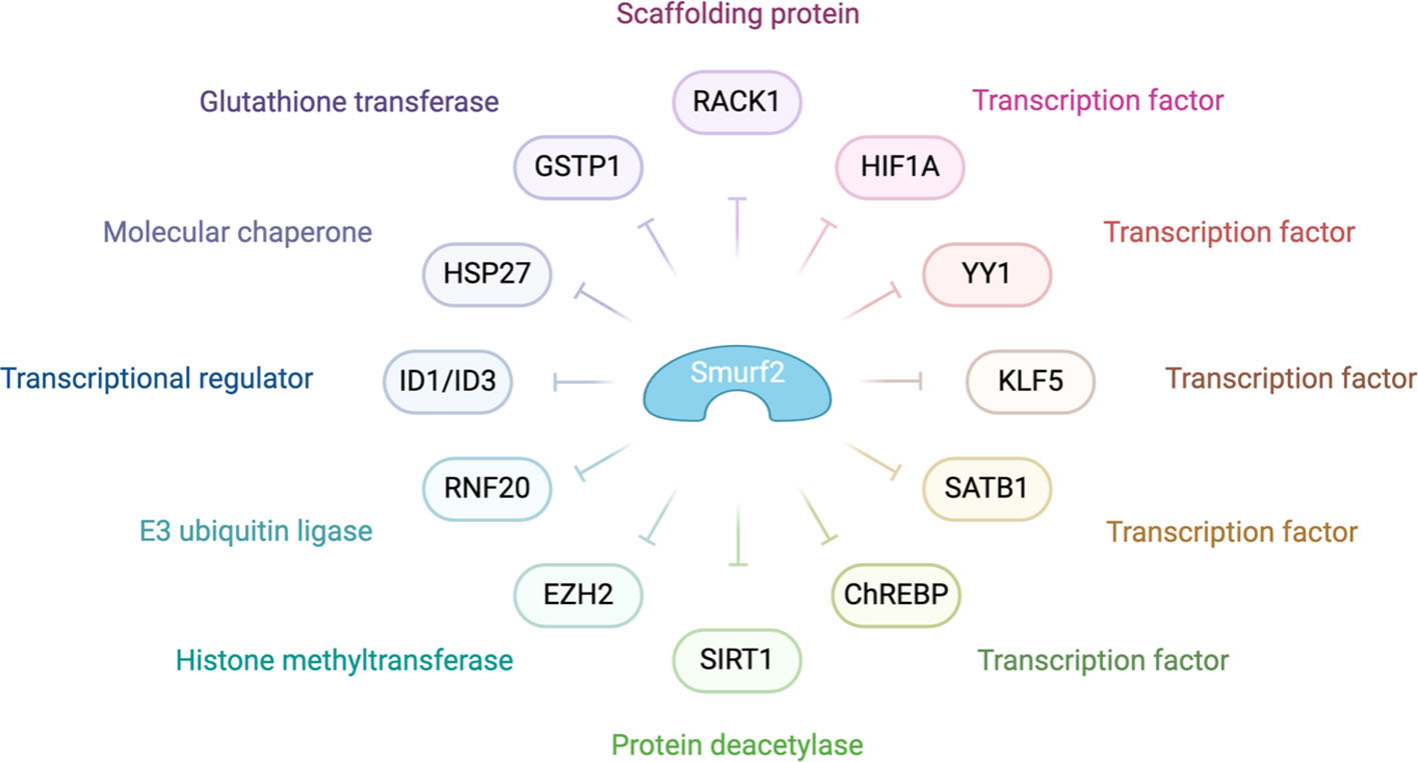
Molecular mechanisms behind SMURF2’s role as a tumor suppressor SMURF2 modulates various cellular processes, including gene expression, chromatin structure, and the DNA damage response, primarily through the ubiquitin-proteasome-mediated degradation pathway. In the context of chromatin compaction and gene regulation, SMURF2 is involved in the degradation of transcription factors such as YY1 and the histone-modifying enzyme RNF20. The interaction with transcriptional regulators like ID1/ID3, mentioned in the diagram, highlights SMURF2’s role in gene expression modulation. Additionally, as noted in the diagram, SMURF2’s regulatory influence extends to other transcription factors and enzymes, playing a critical role in cellular mechanisms that impact tumor suppression and cancer progression (132).

**Figure 4** highlights the pivotal role of SMURF2 in tumor suppression. It achieves this by managing chromatin condensation, DNA damage response, and gene expression through the targeted degradation of key proteins such as the RING-type E3 ubiquitin ligase and RNF20, a histone H2B-modifying enzyme. In addition, SMURF2 governs the proteasomal turnover of proteins like KLF5, ID1/ID3, and YY1. This control impacts tumor biology by modulating p53 activation, suppressing c-Myc, and stabilizing the Mad2 protein, thereby influencing cell division and tumorigenesis (3).

As depicted in **Figure 4**, SMURF2 curbs aerobic glycolysis and cell proliferation in colorectal cancer cells by promoting the ubiquitination and degradation of the carbohydrate response element-binding protein (ChREBP). This interaction between SMURF2 and ChREBP presents a potential target for colorectal cancer management (133). Moreover, SMURF2, an E3 ubiquitin ligase, interacts with the NAD-dependent deacetylase sirtuin 1 (SIRT1), leading to its ubiquitination and degradation. A depletion of SMURF2 results in an upregulation of SIRT1, which fosters the formation and growth of colorectal cancer both *in vitro* and *in vivo*. This finding of a negative correlation between SIRT1 and SMURF2 expression in human colorectal cancer unveils a new potential mechanism for colorectal tumorigenesis via SIRT1 regulation by SMURF2, potentially opening new avenues for colorectal cancer treatment (134).

### 14.2 Advanced insights and therapeutic implications of SMURF2

Recent findings shed light on the intricate involvement of SMURF2 in cancer progression. Specifically, elevated mRNA levels of SMURF2 have been observed to correlate with improved outcomes in clear cell renal cell carcinoma (ccRCC), suggesting a protective function it serves by destabilizing HIF1α. Notably, the heightened levels of SMURF2 also show a positive correlation with enhanced survival in ccRCC, thereby highlighting its potential utility as a biomarker (57). The ability of SMURF2 to regulate protein stability through post-translational modifications underscores its versatility as a promising therapeutic target.

### 14.3 SMURF2’s broad impact across cancer types

SMURF2’s regulatory activities extend across multiple cancers, including NSCLC, where it influences chemotherapy and radiation resistance, demonstrating its importance across various cancer types and treatment scenarios (135). SMURF2’s modulation of HIF1α levels offers a strategic method to interfere with tumors’ hypoxia-driven survival mechanisms.

Additionally, its interactions with TGF-β and RhoA signaling pathways provide a supplementary approach to cancer treatment by affecting hypoxic responses and cellular activities (136). Data from several clinical research revealed that in breast cancer, HIF1α’s role is particularly pronounced. Studies reveal that HIF1α and its transcriptional targets, such as VEGF-A and Hexokinase-I, significantly contribute to angiogenesis and metabolic adaptation. These elements are notably upregulated in hypoxic zones of solid tumors, fostering the aggressive and metastatic potential of tumors. The research further indicates that HIF1α expression is notably higher in triple-negative breast cancer (TNBC) and HER2-positive subgroups, suggesting its role in tumor aggressiveness and poor prognosis (57).

Furthermore, the impact of hypoxia on the immune response within the TME, particularly in TNBC, is evident, as shown by Ma and colleagues. It highlights that hypoxia suppresses the expression of immune effector genes in T and NK cells, leading to immune dysfunction and resistance to immunotherapy. The mechanism involves HIF1α interacting with HDAC1 and PRC2, resulting in chromatin remodeling and epigenetic suppression of effector genes. Targeting HIF1α and associated epigenetic machinery can reverse immune dysfunction and overcome resistance to Programmed Death-1 (PD-1) blockade, as demonstrated in both *in vitro* and *in vivo* models. This identifies a HIF1α-mediated epigenetic mechanism in immune dysfunction and proposes a potential strategy to overcome immune resistance in TNBC (137).

### 14.4 Enhanced stratification and biomarker potential of HIF1α

The expression levels of HIF1α vary significantly among cancers, indicating its utility in patient stratification. High levels often suggest a more aggressive tumor behavior, resistance to therapies, and poorer prognosis. Stratifying patients based on HIF1α expression could enhance disease outcome predictions and treatment responses. This is critical as targeting HIF1α signaling pathways is a focus of ongoing research to develop novel cancer treatments. HIF1α is pivotal in predicting treatment responses and disease outcomes, enhancing adaptability to hypoxic conditions that contribute to tumor progression and resistance (138, 139). This expression influences metabolic pathways and tumor survival under hypoxic conditions, particularly in cancers like NSCLC and pancreatic cancer, where it regulates glucose metabolism and aerobic glycolysis, respectively. Furthermore, HIF1α is implicated in mechanisms such as DNA repair, contributing to chemo- and radio-resistance across various tumors, including glioblastoma, hepatocarcinoma, and lung cancer (60, 69, 138, 140).

Notably, elevated HIF1α levels are strongly correlated with aggressive tumor characteristics such as metastasis, angiogenesis, and resistance to therapy, reflecting its role in the transcriptional regulation of genes crucial for adaptation to hypoxic TME (141). Polymorphisms in the HIF1A gene, such as rs11549465 (1772 C/T) and rs11549467 (1790 G/A), have been linked to an increased susceptibility to various cancers, including prostate cancer and transitional cell carcinoma of the bladder, highlighting their potential as stratification factors for assessing individual cancer risk (142). Interestingly, and as mentioned above, the observation that increased levels of SMURF2 exhibit a favorable association with improved survival in clear cell renal cell carcinoma (ccRCC), thereby emphasizing its potential value as a biomarker (12, 13).

Furthermore, the pivotal role of SMURF2 in the pathogenesis of ovarian cancer underlines its essential function as an E3 ubiquitin ligase for RACK1. This relationship is critical for regulating numerous signaling pathways, where SMURF2’s activity precipitates the polyubiquitination and subsequent destabilization of RACK1. This action unveils a reciprocal expression pattern between SMURF2 and RACK1, with ovarian cancer manifestations displaying lower SMURF2 levels correlating with diminished RACK1 ubiquitination (18). This correlation suggests enhanced RACK1 stability, exacerbating cancer progression and adversely affecting patient prognosis (143). Such findings indicate that the SMURF2-RACK1 axis may be a promising target for therapeutic intervention in ovarian cancer.

While SMURF2 is recognized as a tumor suppressor, its expression in cancer cells varies depending on the type and stage of cancer. This variability presents therapeutic opportunities, where targeting SMURF2 could either amplify its tumor-suppressive functions or mitigate its tumor-promoting activities. Further research is essential to fully understand the mechanisms of SMURF2 and their implications for cancer treatment.

The functions of HIF1α and SMURF2 across different cancer types highlight their significance as therapeutic targets. Continued exploration of the SMURF2-HIF1α pathway will remain a pivotal focus in oncology, with the potential to transform cancer therapy and set new standards in patient care.

## 15 Advancements in SMURF2 and HIF1α interactions: implications for cancer therapy and resistance mechanisms

Recent findings from the El-Deiry lab have provided insight into novel, non-traditional pathways affecting HIF1α stability, thereby highlighting the therapeutic potential of SMURF2. Their research investigates the role of CDKs in regulating HIF1α independent of the von VHL protein, traditionally known to mediate the degradation of HIF1α in response to oxygen levels. This represents a significant departure from established mechanisms, suggesting new avenues for therapeutic intervention (13, 27, 28, 31, 71, 84, 144, 145). The El-Deiry lab observed that CDK1 and CDK4/6 contribute to the stabilization of HIF1α in a manner that is independent of VHL, hypoxia, or p53 status. This suggests that cyclin-dependent kinases regulate HIF1α under normoxic and hypoxic conditions. The utilization of CDK4/6 inhibitors, such as palbociclib, has demonstrated a novel approach to reduce HIF1α levels and impair the hypoxic response in tumors.

### 15.1 Proteomic insights and the role of SMURF2

Further investigation involved a proteomic screen on HIF1α immunoprecipitated from hypoxic colorectal cancer cells treated with CDK inhibitors. The screen revealed that SMURF2, a SMAD-specific E3 ubiquitin-protein ligase, was enriched in cells treated with the CDK4/6 inhibitor palbociclib, highlighting SMURF2’s crucial role in the ubiquitination and subsequent degradation of HIF1α. Knockdown experiments further demonstrated that reducing SMURF2 levels increased basal HIF1α expression, even in CDK inhibitors. Conversely, overexpression of SMURF2 not only inhibited HIF1α expression but also enhanced its ubiquitination under normoxic conditions, indicating a robust mechanism for HIF1α regulation via SMURF2.

### 15.2 Clinical implications and patient outcomes

This research also highlights that higher expression levels of SMURF2 mRNA correlate with improved disease-free survival and overall survival in patients with ccRCC. This correlation points to the potential utility of SMURF2 as both a prognostic biomarker and a therapeutic target in cancer. Building upon our understanding of the SMURF2-HIF1α pathway, recent investigations by the El-Deiry lab have uncovered novel regulatory mechanisms involving cyclin-dependent kinases (CDKs) in HIF1α stability, independent of traditional VHL pathways. This pioneering work highlights the potential of targeting CDKs with specific inhibitors like palbociclib to destabilize HIF1α, offering fresh avenues to impede the hypoxic response integral to tumor survival and proliferation. Further proteomic analyses have **revealed** the significance of SMURF2, a SMAD-specific E3 ubiquitin-protein ligase, which emerges prominently in CDK-inhibited environments.

This finding underscores SMURF2’s role in ubiquitinating and degrading HIF1α, highlighting its therapeutic potential. Gene manipulation studies, including knockdown and overexpression, confirm SMURF2’s regulatory effect on HIF1α levels and its influence under different oxygen conditions. Clinically, higher SMURF2 expression is associated with better outcomes in ccRCC, suggesting its utility as a therapeutic target and prognostic marker.

## 16 Conclusions: harnessing the SMURF2-HIF1α pathway for cancer treatment

The SMURF2-HIF1α pathway presents a compelling target in cancer therapy, given its significant role in regulating hypoxia-driven processes that support tumor survival, angiogenesis, and metabolic adaptation. SMURF2, as an E3 ubiquitin ligase, influences HIF1α degradation independently of the VHL pathway, offering an alternative mechanism for modulating HIF1α activity in hypoxic tumors. Enhancing SMURF2’s function to destabilize HIF1α could disrupt essential adaptive responses within the TME, limiting processes such as the Warburg effect and enabling cancer cells to be more susceptible to conventional therapies like chemotherapy and radiotherapy. This targeting is particularly valuable in aggressive cancers where hypoxic adaptations drive resistance and metastasis, underscoring the therapeutic versatility of SMURF2 as both a tumor suppressor and regulator of hypoxic responses.

Recent insights into SMURF2’s regulatory influence on cellular processes, including chromatin structure, DNA damage response, and protein degradation, extend its therapeutic relevance beyond HIF1α. This regulatory breadth positions SMURF2 as a critical modulator of tumor progression and resistance. For instance, SMURF2-mediated degradation of oncogenic factors not only limits cancer cell proliferation but also enhances the efficacy of DNA-damaging treatments by reducing cancer cells’ repair capabilities. The duality of SMURF2’s functions in cancer—acting both to suppress and support tumor development depending on cellular context—offers an opportunity for tailored interventions that exploit these mechanisms selectively.

Future therapeutic strategies should focus on developing SMURF2-specific modulators capable of fine-tuning HIF1α stability under hypoxic conditions, potentially enhancing the disruption of the TME. Such targeted approaches could inhibit the angiogenic and metabolic dynamics that promote tumor resilience and adaptation, particularly in cases resistant to existing treatments. These modulators could selectively impair HIF1α-driven metabolic reprogramming and angiogenesis, two hallmark features of hypoxic tumors, which are essential for cancer cell survival and invasiveness.

Harnessing the SMURF2-HIF1α pathway also opens doors to combinatorial treatments. By incorporating SMURF2-based interventions with CDK4/6 inhibitors or heat shock protein HSP-90 inhibitors, there is potential to synergize effects on HIF1α degradation, disrupting cancer cells’ hypoxic adaptations more robustly. Furthermore, SMURF2’s recently discovered role in promoting ferroptosis by targeting GSTP1 degradation introduces another promising avenue, where SMURF2 modulators could induce ferroptotic cell death in therapy-resistant cancers. This ferroptosis pathway, acting independently of traditional apoptosis mechanisms, could offer an alternative to circumventing cancer cells’ resistance to apoptosis.

Despite the promising potential, targeting the SMURF2-HIF1α pathway presents specific challenges. Modulating a pathway that significantly impacts both tumorigenic and physiological processes carries inherent risks, including potential off-target effects that could interfere with normal cellular functions such as erythropoiesis and cellular response to hypoxia. Therefore, the development of selective SMURF2 inhibitors that avoid unintended interactions and minimize systemic toxicity is crucial. Additionally, because cancer cells exhibit high genetic plasticity, the risk of resistance development to SMURF2-targeted therapies remains a concern, underscoring the need for ongoing research into combination therapies that prevent or counteract adaptive resistance.

To bring the SMURF2-HIF1α pathway closer to clinical applications, future research must also address the practical aspects of delivery and pharmacokinetics of SMURF2 modulators. Optimizing these drugs to ensure effective delivery to tumor sites, alongside clinical trials to evaluate safety and efficacy, will be essential for successful implementation. Additionally, economic considerations, including research and development costs, must be factored into strategic planning to make these potential therapies accessible to patients. The SMURF2-HIF1α axis thus represents a new frontier in cancer therapy with the potential to reshape treatment paradigms for hypoxia-adapted tumors. Targeting this pathway holds promise for enhancing cancer therapy, improving patient outcomes, and providing durable responses by weakening the hypoxic adaptations that allow tumors to evade conventional treatments. Continued research to elucidate the molecular intricacies within the SMURF2-HIF1α pathway, coupled with innovative drug design, could unlock highly effective, targeted therapeutic strategies that transform cancer management, particularly in resistant and aggressive cancer types
